# Improvement of Surgical Wound Healing With Ozonated Oil in Bilateral Breast Surgery: A Pilot Intra-patient Comparative Study

**DOI:** 10.7759/cureus.85829

**Published:** 2025-06-12

**Authors:** Alejandro Ruiz-Vall, Ana Altamirano-Faus, Nerea Bedmar-Gonzalez, Juan Jose Andres-Lencina

**Affiliations:** 1 Plastic Surgery, Ilahy Gandia, Gandia, ESP; 2 Dermatology, Hospital Vega Baja, Alicante, ESP

**Keywords:** breast surgery, ozonated oil, postoperative care, scar management, wound healing

## Abstract

Objective: To evaluate the efficacy of ozonated oil compared to conventional wound care in enhancing postoperative wound healing and cosmetic outcomes in bilateral breast surgery. The primary outcomes were pruritus, pain, and scar aesthetics (color, texture, and thickness) at six weeks.

Methods: A prospective, self-controlled pilot study was conducted on five female patients undergoing bilateral breast surgery (10 wounds). One breast received conventional care (cleaning with saline and topical antiseptics), while the contralateral breast was treated with topical ozonated oil (Ozoaqua®, Spain). Pain, pruritus, and scar appearance (color, texture, and thickness) were evaluated at six weeks using patient-reported scales (0-10). A blinded physician independently evaluated scar quality. Data were analyzed using paired t-tests and Wilcoxon tests.

Results: Wounds treated with ozonated oil demonstrated significantly lower pain (2.4 ± 1.1 vs. 4.0 ± 1.2; p = 0.011), reduced pruritus (median score 1 vs. 3; p = 0.042), and superior scar color (4.2 ± 0.8 vs. 3.4 ± 0.5; p = 0.05), texture (4.4 ± 0.5 vs. 3.2 ± 0.8; p = 0.03), and thickness (4.0 ± 0.7 vs. 3.0 ± 1.0; p = 0.04). The blinded evaluator favored ozonated oil-treated scars in four out of five cases.

Conclusions: Ozonated oil significantly enhanced wound healing outcomes, reducing pain and pruritus and improving scar aesthetics compared to conventional care. These findings support further investigation through larger randomized trials. Given its low cost and ease of use, ozonated oil may be a valuable adjunct in rural plastic surgery settings where access to postoperative scar management resources is limited.

## Introduction

Cosmetic and oncologic breast surgeries aim to minimize conspicuous scarring and enhance patient satisfaction. Despite advancements in surgical technique, many patients still experience suboptimal scar outcomes or prolonged healing. Ozone-based therapies, delivered topically via ozonized oils, are suggested to stimulate the wound-healing cascade by modulating local inflammation, enhancing antimicrobial control, and promoting tissue repair. Ozonated vegetable oils act as controlled-release systems for low concentrations of reactive oxygen species. When applied to tissue, the peroxides formed during ozonation break down into molecular oxygen and a cascade of lipid-derived mediators that support healing in three ways. First, they modulate the local cytokine milieu, down-regulating pro-inflammatory signals such as TNF-α and IL-1β while stimulating growth factors that foster fibroblast proliferation and collagen deposition. Second, the transient oxygen burst improves micro-circulatory perfusion and raises tissue pO₂, accelerating re-epithelialization and angiogenesis. Third, the peroxidized lipids display broad antimicrobial activity against common skin pathogens, reducing bioburden without promoting antibiotic resistance. This multifaceted action provides a biological rationale for testing ozonated oil as an inexpensive postoperative adjunct [1,2].

Although ozone therapy has demonstrated benefits in chronic ulcers and burn wounds [3], its impact on elective surgical scars, particularly in aesthetic procedures, remains insufficiently explored. Given the symmetrical nature of bilateral breast operations, each patient can serve as an internal control to compare standard care versus adjunct therapy on matched incisions. The limited availability of specialized scar management and follow-up in rural plastic surgery practice was recently highlighted by Koenig et al. [4]. Low-cost topical adjuncts that patients can self-apply, such as ozonated oil, could, therefore, be particularly impactful outside major referral centers. We hypothesized that adding topical ozonized oil to conventional care would improve wound healing outcomes and patient satisfaction. This self-controlled study was undertaken to gather preliminary data and inform the design of future, larger trials.

## Materials and methods

Study design and setting

We conducted a prospective, self-controlled pilot study at the Ilahy Aesthetic Surgery Unit, IMED Gandía, Spain, between December 2022 and February 2023. The study followed the principles of the Declaration of Helsinki (2013 revision). Due to its minimal-risk nature, our institutional review board waived formal ethics review. All participants provided written informed consent for data collection and publication of de-identified images.

Participants

Five female patients (aged 32-58 years) undergoing symmetrical bilateral breast procedures (either aesthetic or oncologic) were identified from chart reviews. Eligible patients had two comparable surgical incisions, one per breast, placed in a similar location (e.g., inframammary fold). Exclusion criteria included uncontrolled diabetes, active infection, known allergy to the oil, or other adjunctive scar therapies (e.g., silicone gel) on either incision. Every participant consented to have one side treated with topical ozonized oil (Ozoaqua®, Spain) in addition to standard wound management, while the other side (control) received standard care alone.

Intervention and side allocation

Standard wound care involved twice-daily cleaning with water, glycerin soap, and chlorhexidine solution. All ten incisions were made by the same board-certified plastic surgeon with over 10 years of experience.

The ozonated oil treatment followed the same cleaning regimen, with the addition of topical ozonated oil applied along the incision line and gently massaged twice daily for approximately three weeks. The ozonated oil was donated free of charge by the manufacturer to both the center and the patients.

Allocation of ozonized oil to the left or right breast was randomized by each patient (e.g., coin flip). Surgeons and nursing staff knew which side had ozonized oil, but the independent evaluator for scar outcomes was blinded to the assignment.

Outcome measures

Evaluations occurred around six weeks post-surgery (range six to eight weeks) after incisions had epithelialized. Two main assessments were performed. Patient-reported outcomes included pruritus (itch) and pain, each rated on a 0-10 scale, where 0 indicated no symptom and 10 represented the worst imaginable. Scar appearance was evaluated based on color, texture, and thickness, with each parameter rated from 0 to 10, where 0 indicated the poorest possible appearance and 10 indicated a match with normal skin. Overall scar satisfaction was also rated on a 0-10 scale, with 0 representing not at all satisfied and 10 representing extremely satisfied. In the independent medical evaluation, a plastic surgeon reviewed standardized photographs of both incisions, which were presented in random order. The global scar outcome was rated on a 0-10 scale, with higher scores indicating better appearance.

Statistical analysis

Normality Testing and Choice of Statistical Tests

Differences (ozonized side minus control side) for each variable (pruritus, pain, scar measures, and satisfaction) were assessed via the Shapiro-Wilk test. When differences were normally distributed (p>0.05), a paired t-test was used; otherwise, a Wilcoxon signed-rank test was employed [5]. The small sample size (n = 5) prompted careful use of these methods.

Significance Threshold, Effect Sizes, and Main Results

A two-sided α = 0.05 defined statistical significance. For paired t-tests, Cohen’s d for repeated measures was calculated (mean of differences / SD of differences) [6]. For Wilcoxon, we used r = (Wilcoxon Z) / √(number of observations). Interpreting effect sizes followed standard conventions (|d|≥0.8 or |r|≥0.5 = large). SPSS v27 (IBM Corp., Armonk, NY) and GraphPad Prism 9 (GraphPad Software, San Diego, CA, USA) aided computations.

## Results

Participant characteristics

**Figure 1:**
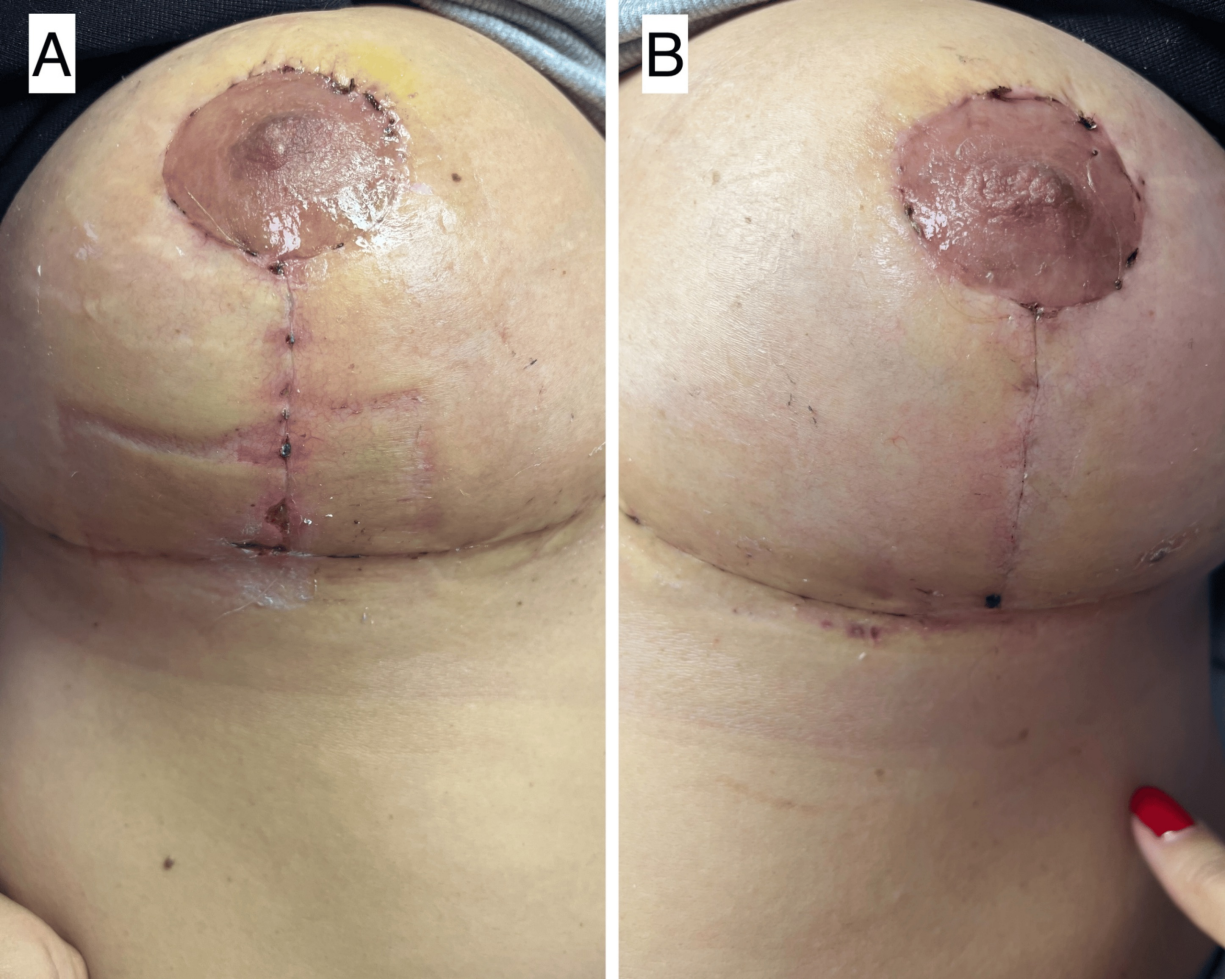
Postoperative comparison in a patient after bilateral breast augmentation. (A) Right breast scar treated with standard wound care (glycerin soap and chlorhexidine). (B) Left breast scar treated with ozonized oil, showing improved healing with reduced crusting, more uniform pigmentation, and flatter scar contour. Photographs taken four weeks after surgery.

**Figure 2:**
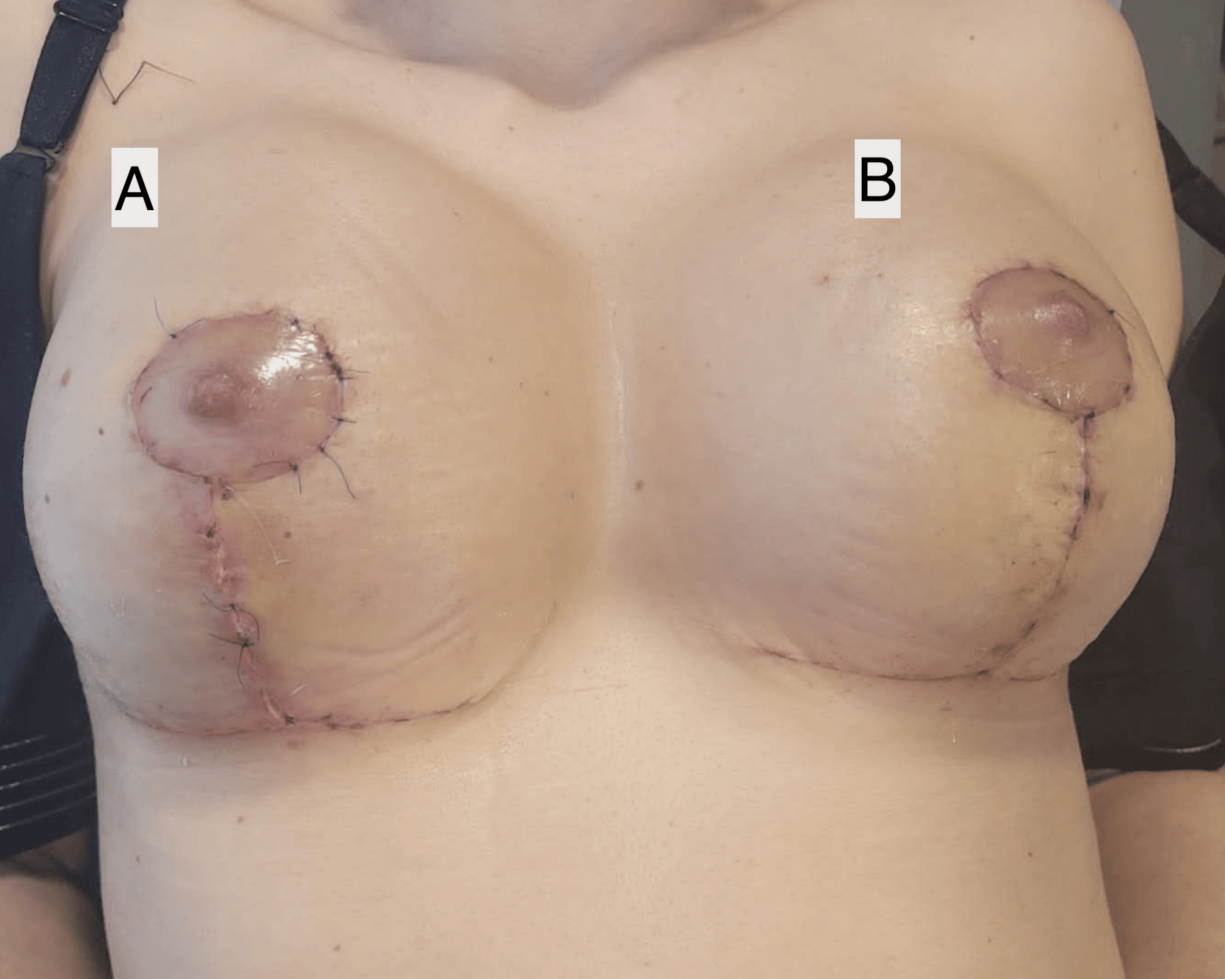
Postoperative outcome in another patient undergoing bilateral breast augmentation. (A) Left breast treated with standard care. (B) Right breast treated with ozonized oil, showing improved scar integration and less edema. Note the persistence of residual sutures in the control side (left), which had not yet been fully removed at the time of follow-up. Photographs taken three weeks postoperatively.

All five patients completed the protocol, yielding 10 incision sites. The five patients underwent bilateral aesthetic procedures (augmentation/reduction). No adverse events, infections, or wound dehiscences were reported. Healing proceeded normally on both sides, with full epithelial closure achieved by approximately 2 weeks postoperatively, as shown in the accompanying images (Figure 1 and Figure 2).

Results of the statistical analysis

Verification of Normality

Shapiro-Wilk tests indicated that the differences in pruritus, scar color, texture, thickness, and patient satisfaction did not significantly deviate from normality (p > 0.05). However, the differences in pain and independent evaluator satisfaction were non-normally distributed (p < 0.01). Therefore, paired t-tests were used for normally distributed differences, and Wilcoxon signed-rank tests were applied for non-normal differences.

Significance, Effect Sizes, and Main Findings

The results are summarized in Table 1.

**Table 1:** Comparison of outcomes between ozonized oil and conventional care for surgical breast scars. Data presented as mean ± SD. ^a^Analyzed via the Wilcoxon signed-rank test due to non-normal distribution; all other outcomes were analyzed using paired t-tests. Bolded p-values indicate statistically significant differences (p < 0.05). Effect sizes are considered large for |d| ≥ 0.8 or r ≥ 0.5. Score interpretations: Pruritus and pain (0-10): 0 indicates “no symptoms,” and 10 represents the “worst imaginable symptoms.” Lower scores reflect better clinical outcomes. Scar color, texture, and thickness (0-10): Higher scores indicate better scar appearance, resembling normal skin. Satisfaction (patient and evaluator, 0-10): 0 indicates “not satisfied at all,” and 10 indicates “completely satisfied.”

Outcome	Ozonized (Mean ± SD)	Conventional (Mean ± SD)	Test Statistic	P-value	Effect Size	95% CI Difference
Pruritus (0-10)	3.0 ± 1.2	6.2 ± 2.2	t(4) = -3.00	0.040	d = -1.34	(-6.16, -0.24)
Pain (0-10)^a^	3.8 ± 1.6	4.6 ± 1.5	W = 0	0.317	r = 1.00	–
Scar Color (0-10)	8.8 ± 0.4	7.2 ± 0.8	t(4) = 3.14	0.035	d = 1.40	(0.18, 3.02)
Scar Texture (0-10)	9.0 ± 0.0	7.6 ± 1.1	t(4) = 2.75	0.052	d = 1.23	(-0.02, 2.82)
Scar Thickness (0-10)	8.8 ± 0.4	7.4 ± 0.9	t(4) = 2.75	0.052	d = 1.23	(-0.02, 2.82)
Patient Satisfaction (0-10)	8.8 ± 0.4	7.4 ± 0.9	t(4) = 2.75	0.052	d = 1.23	(-0.02, 2.82)
Evaluator Satisfaction (0-10)^a^	9.0 ± 0.0	7.6 ± 0.5	W = 0	0.062	r = 0.90	-

Despite a very small sample, effect sizes indicate a positive impact of ozonized oil on scar-related outcomes, especially in reducing pruritus and improving color. Pain relief did not differ between treatments.

## Discussion

Our findings suggest that the addition of ozonized oil to standard postoperative wound care can enhance scar appearance, particularly regarding scar color, and reduce associated pruritus in patients undergoing bilateral breast surgery, thereby improving overall patient satisfaction. These clinical observations are consistent with existing evidence indicating that ozonated products can beneficially modulate inflammatory responses, improve local blood circulation, and provide antimicrobial control [1,3,7,8].

In a relevant preclinical investigation, Valacchi et al. (2011) demonstrated in murine models that ozonated sesame oil significantly accelerated the wound-healing process through mechanisms involving enhanced re-epithelialization, improved collagen fiber organization, and reduced inflammation [7]. These mechanisms may directly underpin the clinical improvements observed in our patients, particularly in scar appearance parameters such as color and texture.

Further supporting our observations, Schreml et al. (2010) emphasized that adequate oxygenation is a critical determinant of wound-healing quality, particularly during the proliferative and remodeling phases, promoting faster and more organized tissue repair [9]. Given ozone’s established role in enhancing local tissue oxygen availability by stimulating oxygen metabolism, improved scar maturation observed in our study might indeed reflect optimized local oxygen conditions fostered by ozonized oil application.

Moreover, the reduction of pruritus noted in our study aligns with earlier reports suggesting that ozonated products could alleviate local inflammatory symptoms and discomfort associated with wound healing [3,7,8]. While pain perception remained unchanged between the treatment groups in our study, this finding was expected due to uniform postoperative analgesic protocols. However, the substantial effect size observed in pruritus reduction highlights ozone’s additional potential benefit, particularly relevant in postoperative care aimed at improving patient comfort and quality of life during the healing phase.

No infections were documented within our sample, a finding consistent with previous studies demonstrating that ozonated sunflower oil (Oleozon) exhibits significant antimicrobial effects against common skin pathogens such as Staphylococcus aureus and Pseudomonas aeruginosa [1]. Given these antimicrobial properties, it would be of clinical interest to investigate in larger studies whether ozonized oil might further reduce wound-related complications such as infection and prolonged inflammatory responses, particularly in high-risk surgical populations.

Despite the encouraging results, our study presents certain limitations. Primarily, the small sample size (n = 5) significantly limits statistical power and generalizability. Furthermore, some outcomes, such as scar texture, thickness, and satisfaction (both patient and evaluator), showed trends toward significance but did not reach conventional statistical thresholds (p-values just above 0.05). Formal randomization software was not used. Larger studies would be beneficial to clarify the magnitude and clinical relevance of these observations. Additionally, our follow-up duration (~6 weeks) may not capture the complete remodeling phase of scar formation, which typically evolves over months to years postoperatively. 

Future research should thus employ prospective, randomized controlled designs involving larger patient cohorts and extended follow-up periods to verify and extend our preliminary findings. Incorporating validated scar assessment scales, such as the Patient and Observer Scar Assessment Scale (POSAS), would facilitate more accurate quantification of both subjective and objective scar characteristics and patient satisfaction, thereby allowing more precise and reliable comparisons across studies (Draaijers et al., 2004) [10]. Moreover, assessing specific biological markers of inflammation, oxidative stress, and collagen synthesis could further elucidate the underlying molecular mechanisms by which ozonized oil exerts its beneficial effects in wound healing.

This has broader clinical relevance, particularly in rural practice, where plastic surgery services often lack ready access to silicone dressings, laser therapy, or specialist nurse follow-up [10]. Our findings suggest that a simple, inexpensive product such as ozonated oil could partially bridge this gap, empowering patients to self-manage scars when regular reviews are impractical. Future trials should include rural patient cohorts and cost-effectiveness analyses.

In conclusion, despite methodological limitations, the consistent direction and magnitude of our results suggest that topical ozonized oil could represent a valuable adjunctive therapy in postoperative wound care, particularly for enhancing aesthetic outcomes and reducing associated pruritus. Further robust studies are warranted to substantiate these promising findings and potentially expand clinical guidelines regarding postoperative scar management.

## Conclusions

Topical ozonized oil showed promising improvements in scar color, pruritus, and overall satisfaction in this pilot. While preliminary, these findings warrant larger controlled trials with extended follow-up. If confirmed, ozonized oil may be a safe, cost-effective option for enhancing post-surgical healing and cosmetic results in breast operations.
